# Integrated Metabolomic and Bioreporter Screening of Five Thai Mushroom Extracts: Exploring Antibiofilm and Biocompatibility Profiles

**DOI:** 10.3390/molecules31193457

**Published:** 2026-09-28

**Authors:** Yardnapar Parcharoen, Calin Trif, Jovana Vunduk, Robert S. Marks, Chiravoot Pechyen, Muhammad Danial Che Ramli, Aporn Bualuang

**Affiliations:** 1 Chulabhorn International College of Medicine, Thammasat University, Pathum Thani 12120, Thailand; yardnapa@tu.ac.th; 2 Department of Materials Technology and Textile, Faculty of Science and Technology, Thammasat University, Pathum Thani 12120, Thailand; ict_temp724@tu.ac.th (C.T.); cpechyen@tu.ac.th (C.P.); 3 Thammasat University Center of Excellence in Modern Technology and Advanced Manufacturing for Medical Innovation, Thammasat University, Pathum Thani 12120, Thailand; 4 Institute of General and Physical Chemistry, Studentski trg 12/V, 11158 Belgrade, Serbia; jvunduk@iofh.bg.ac.rs; 5 Avram and Stella Goldstein-Goren Department of Biotechnology Engineering, Faculty of Engineering Sciences, Ben-Gurion University of Negev, Beer Sheva 84105, Israel; rsmarks@bgu.ac.il; 6 Department of Diagnostic and Allied Health Science, Faculty on Health and Life Sciences, Management and Science University, Shah Alam 40100, Selangor, Malaysia; 7 Neuroscience and Mental Wellbeing (NeuroMIND), Management and Science University, Shah Alam 40100, Selangor, Malaysia

**Keywords:** Thai mushrooms, *Termitomyces clypeatus*, *Lentinus squarrosulus*, antimicrobial synergism, LC-QTOF-MS, bioluminescent bioreporter, HaCaT biocompatibility

## Abstract

This study evaluated the biomedical potential of ethanolic extracts from five native Thai mushrooms, revealing distinct biological activities associated with their metabolomic profiles. *Termitomyces clypeatus* R. Heim demonstrated exceptional biocompatibility, effectively promoting human keratinocyte (HaCaT) proliferation without causing cytotoxicity to the cells. Its primary metabolome, rich in essential amino acids and nucleosides, supports its potential as a candidate for topical wound healing, pending further functional validation. Conversely, *Lentinus squarrosulus* exhibited strong antibiofilm properties and moderate direct antimicrobial activity against *Staphylococcus aureus* and *Pseudomonas aeruginosa*, demonstrating a weak additive effect with gentamicin. Mechanistic bioreporter screening and Liquid Chromatography Quadrupole Time-of-Flight Mass Spectrometry (LC-QTOF-MS) suggested that this efficacy may be associated with quorum-sensing disruption and the presence of bioactive hydroquinolones, in addition to potential genotoxic stress induction. While *Schizophyllum commune* displayed moderate effects, *Volvariella volvacea* and *Thaeogyroporus porentosus* exhibited high-dose cytotoxicity linked to severe oxidative stress and high organic acid content, limiting their direct high-dose biomedical applications. Ultimately, *T. clypeatus* R. Heim and *L. squarrosulus* have emerged as promising candidates that warrant further investigation for tissue regeneration and potential adjunctive anti-infective applications.

## 1. Introduction

The rising incidence of chronic wounds complicated by biofilm-associated bacterial infections represents a critical and escalating challenge in modern biomaterials science and clinical management. Over 90% of chronic wounds harbor opportunistic pathogens, such as *Staphylococcus aureus* (*S. aureus*) and *Pseudomonas aeruginosa* (*P. aeruginosa*), which colonize compromised dermal barriers by establishing robust extracellular polymeric substance (EPS) matrices [1,2]. These mature biofilms physically shield the embedded pathogens, facilitate the exchange of resistance genes, and actively neutralize conventional antimicrobial agents, leading to recalcitrant infections and severe delays in tissue regeneration [2]. Consequently, the development of next-generation topical biomaterials necessitates dual-action therapeutic platforms, such as engineered systems or active agents capable of potent, targeted antibiofilm efficacy without exhibiting cytotoxicity toward host mammalian cells, such as human keratinocytes [3,4].

To circumvent the accelerating global crisis of antimicrobial resistance, there has been a significant shift toward harnessing complex natural metabolomes to disrupt bacterial communication and structural integrity, rather than relying solely on traditional, highly selective bactericidal mechanisms. Macrofungi serve as prolific biological factories, synthesizing a vast array of bioactive secondary metabolites, including complex alkaloids, triterpenoids, hydroquinolones, and phenolic compounds [5]. Certain fungal-derived molecules have shown promise in penetrating biofilm structures and actively blocking the quorum sensing (QS) signaling pathways that are essential for biofilm formation [6]. While the nutritional value of native tropical mushrooms is well documented, their precise biomedical utility as a source of synergistic antimicrobial and tissue-regenerative agents remains largely unexplored.

Despite the growing clinical interest in fungal-derived therapeutics, a significant knowledge gap persists regarding their exact molecular mechanisms of action. Most biological evaluations of fungal extracts are confined to fundamental phenotypic observations, such as determining minimum inhibitory concentrations (MIC) [7]. To translate these natural resources into engineered biomaterials or synergistic clinical therapies, it is imperative to elucidate how these complex extracts interact with bacterial cells, whether by disrupting QS networks, inducing genotoxic stress, or inhibiting essential biosynthetic pathways, and correlate these mechanistic actions directly with their chemical profiles.

To address this challenge, the present study systematically evaluated the biomedical potential and precise molecular mechanisms of ultrasound-assisted ethanolic extracts from five native Thai mushroom species: *Termitomyces clypeatus* R. Heim (*T. clypeatus* R. Heim), *Volvariella volvacea* (*V. volvacea*), *Schizophyllum commune* (*S. commune*), *Lentinus squarrosulus* (*L. squarrosulus*), and *Thaeogyroporus porentosus* (*T. porentosus*). This study employed a chemistry-driven, multi-tiered screening pipeline to evaluate the suitability of these natural sources for topical application in biomedicine. First, non-targeted Liquid Chromatography Quadrupole Time-of-Flight Mass Spectrometry (LC-QTOF-MS) metabolomic profiling was performed to map the complex chemical composition of each species. Second, the biocompatibility and proliferative potential of the extracts were evaluated using human keratinocytes (HaCaT cells). Subsequently, their antimicrobial efficacy, synergistic interactions with conventional antibiotics, and capacity to inhibit bacterial adhesion and biofilm formation were evaluated. Finally, rather than relying on phenotypic data alone, we utilized a high-throughput panel of six genetically modified bioluminescent *E. coli* bioreporter strains [8] to rapidly map the specific molecular stress pathways induced by the extracts, directly correlating these distinct mechanisms with their chemical compositions. We hypothesized that distinct metabolomic profiles among Thai mushroom species would produce differential antimicrobial, antibiofilm, and biocompatibility activities that could be mechanistically explained using bacterial bioreporter screening.

## 2. Results and Discussion

### 2.1. LC-MS Metabolomic Profiling and Mechanistic Correlation

To elucidate the chemical basis for the observed antimicrobial, antibiofilm, and biocompatibility activities, 70% ethanolic extracts of the five Thai mushroom species were subjected to non-targeted metabolomic profiling using LC-QTOF-MS. Following data filtration and normalization (retention time limit 0.2–18 min, mass error ≤ ±25 ppm, identification score ≥ 0.70), the analysis successfully detected a total of 939 features in the positive ionization mode and 718 features in the negative ionization mode. The metabolomic profiles varied significantly across the five species and were directly correlated with their distinct biological behaviors of the species. A summary of the key identified metabolite classes and their correlated biological activities is presented in Table S1 (Supporting Information).

### 2.2. Keratinocyte Viability and Biomedical Biocompatibility (CCK-8 Assay)

To evaluate the biocompatibility and potential proliferative effects of the five mushroom ethanolic extracts for topical biomedical applications, an in vitro Cell Counting Kit-8 (CCK-8) assay was performed using the HaCaT human keratinocyte cell line. HaCaT cells were obtained from Cell Lines Service GmbH (Eppelheim, Germany). The results, expressed as the percentage of cell viability relative to that of the untreated control, are shown in Figure 1.

In the landscape of topical biomaterials, crude botanical and fungal extracts frequently exhibit dose-limiting toxicity at concentrations exceeding 500 µg/mL because of the non-specific, membrane-lytic action of ubiquitous compounds, such as saponins and tannins [9]. In these circumstances, the biological profile of *T. clypeatus* R. Heim is exceptional. It exhibited a clear, dose-dependent stimulatory effect without any signs of cytotoxicity, significantly increasing viability at 200 µg/mL (*p* < 0.01) and peaking at a significantly mitogenic level, increasing cell viability to 118.82% (*p* < 0.001) at the maximum concentration of 2000 µg/mL. Conversely, *V. volvacea* and *T. porentosus* exhibited a biphasic response and significantly high cytotoxicity at high concentrations (Figure 1). *V. volvacea* stimulated growth at lower doses but caused a significant decrease in viability at 2000 µg/mL (*p* < 0.001). *T. porentosus* exhibited a continuous, dose-dependent inhibitory effect, culminating in a significant reduction in cell viability (83.89%, *p* < 0.01) at 2000 µg/mL.

It is important to acknowledge that these findings are based on a 24 h viability assessment in an immortalized HaCaT keratinocyte line. Although HaCaT cells are a well-established model for initial dermatological screening, they are aneuploid and exhibit proliferation kinetics that may differ from those of primary human keratinocytes [10]. Furthermore, a 24 h viability endpoint provides only a snapshot of cellular health and does not account for long-term cytotoxicity, genotoxicity, or complex functional parameters such as cell migration, re-epithelialization, or extracellular matrix remodeling. Consequently, while *T. clypeatus* R. Heim demonstrates excellent in vitro biocompatibility and proliferative potential, these results remain preliminary. Doubts regarding its wound-healing efficacy remain speculative until validated in physiologically relevant models, such as 3D human skin equivalents, ex vivo skin explants, or in vivo wound models, which better account for tissue stratification, immune cell infiltration, and angiogenesis.

### 2.3. Antimicrobial Activity

The baseline antimicrobial activity of the 70% ethanolic extracts was evaluated by determining the MIC and MBC. As summarized in Table 1, the extracts demonstrated variable strain-specific efficacy. The extracts demonstrated limited direct bactericidal activity against the tested pathogens, including Gram-negative *P. aeruginosa*. However, their antibiofilm efficacy was significant. Despite *P. aeruginosa* possessing a highly restrictive outer membrane and sophisticated multidrug efflux pumps (e.g., MexAB-OprM) that natively exclude many bulky natural products, *T. clypeatus* R. Heim achieved 100% adhesion inhibition [11]. All species, except *V. volvacea*, completely prevented mature biofilm formation at higher concentrations of the mushroom solution. Regarding direct antimicrobial action, *L. squarrosulus* exhibited the most notable efficacy against *S. aureus* (MIC = 2.5 mg/mL and MBC = 20 mg/mL).

The antimicrobial activity observed for these crude extracts required relatively high working concentrations, with MICs in the mg/mL range. Although these values demonstrate baseline bioactivity, such high concentrations present significant translational limitations for clinical formulations, particularly concerning solubility issues and the potential for local tissue irritation. Consequently, these crude extracts are not suitable for direct clinical application as standalone topical antimicrobials. Future investigations should prioritize bioassay-guided fractionation to isolate and purify specific, highly potent active compounds, which is essential for significantly lowering the effective inhibitory concentrations.

### 2.4. Antimicrobial Synergism (Checkerboard Assay)

To evaluate the interaction between mushroom extracts and conventional antibiotics, a checkerboard assay was performed using gentamicin against *S. aureus* ATCC 25923, as most samples exhibited initial MIC values for this specific strain. The mushroom extracts were applied across a concentration range of 0.156–20 mg/mL, whereas gentamicin was applied at concentrations ranging from 0.0039 to 0.5 mg/mL.

The interactive effects of these combinations were quantified using the Fractional Inhibitory Concentration Index (FICI). The outcomes were classified based on standard criteria: synergism (FICI ≤ 0.5), additive effect (0.5 < FICI ≤ 1.5), indifference (1.5 < FICI ≤ 4), and antagonism (FICI > 4) [12].

As detailed in Table 2, most of the tested extracts demonstrated indifferent interactions when combined with gentamicin. *L. squarrosulus* yielded an FICI of 0.832. While standard pharmacological classifications often categorize FICI values between 0.5 and 1.5 as additive, an FICI of 0.832 is near the indifference threshold and represents a weak additive effect rather than the strong synergy (FICI ≤ 0.5) required to support a definitive multi-fold reduction in clinical antibiotic dosage [13,14,15,16]. Further in vivo pharmacokinetic and time-kill kinetic studies are necessary to explore its potential role as a clinical adjuvant.

### 2.5. Preliminary Screening of Antiadhesion and Antibiofilm Efficacy

The detailed raw dataset for bacterial adhesion inhibition at all tested concentrations is provided in Supporting Information Table S3. Briefly, the most profound antiadhesion activity was observed against *P. aeruginosa*, with *T. clypeatus* R. Heim achieving an exceptional 100% inhibition at intermediate concentrations (2.5–10 mg/mL), as shown in Table 3. Other extracts demonstrated variable, non-monotonic dose–response behaviors, highlighting the complexity of crude extract interactions at different concentrations.

Interestingly, the antiadhesion data exhibited non-monotonic sinusoidal dose–response curves, rather than simple linear trends. This complex behavior is a documented phenomenon in natural product chemistry, often attributed to the shifting physicochemical states of secondary metabolites [17]. We hypothesized that at low and intermediate concentrations, the active secondary metabolites effectively block bacterial surface receptors. However, at supersaturating concentrations, amphiphilic molecules (such as long-chain fatty acids) may surpass their critical micelle concentration (CMC) [18]. This physicochemical shift could dynamically alter the surface tension of the polystyrene test wells, potentially creating a micellar scaffold that provides a hydrophobic interface for physical bacterial attachment, thereby masking the true biological inhibitory effect of the extract at the highest concentration.

It is important to emphasize that this CMC hypothesis remains a preliminary interpretation of the observed data and does not constitute definitive proof, as direct surface tension measurements or micellar characterization (e.g., DLS and TEM) were not performed in this study. Furthermore, because crystal violet staining measures total biomass and cannot distinguish between viable cells, dead cells, and the extracellular matrix, the observed reductions should be interpreted strictly as changes in the overall biomass rather than direct evidence of biofilm-cell killing. Additionally, because all tests were conducted exclusively on polystyrene plates, there was a risk of substrate-dependent bias. Therefore, these findings should be regarded as initial descriptive screening. Future studies are essential to decouple these mechanisms, particularly through the use of parallel CFU enumeration to assess viability, live/dead staining (SYTO 9/propidium iodide) for microscopic architectural analysis, and standardized testing across diverse surfaces (glass and tissue culture plastics) to eliminate substrate-dependent confounding effects.

### 2.6. Mechanistic Elucidation via Bioluminescent Bioreporter Screening

To generate preliminary mechanistic hypotheses regarding the antimicrobial and antibiofilm activities of the extracts, high-throughput screening was performed using a panel of six genetically modified *E. coli* bioreporter strains. Although these bioreporters are highly sensitive to specific stress pathways, it is important to acknowledge that they function as heterologous models. The cellular response to the mushroom extracts was quantified using the Induction Factor (IF). In this assay, an IF > 1.5 indicates significant induction of a specific stress pathway, an IF < 0.5 indicates inhibition of bioluminescence (often correlating with pathway suppression or acute lethality), and an IF between 0.5 and 1.5 indicates a non-significant effect [19]. These data serve as an initial screening tool to identify candidate stress-response pathways; however, they do not definitively establish the molecular basis of action in the clinical pathogens *P. aeruginosa* or *S. aureus*, which possess divergent regulatory networks and stress response machinery. Future studies utilizing pathogen-specific assays, such as RT-qPCR of *lasI* or *agrA* expression, will be necessary to validate these preliminary findings. The comprehensive mechanistic profiles of all five extracts across a concentration gradient (0.15–5 mg/mL) revealed highly distinct pathways of action for each mushroom species, as summarized in Figure 2.

#### 2.6.1. Disruption of Quorum Sensing

Quorum sensing is a fundamental cell-to-cell communication network utilized by bacteria to coordinate their virulence and biofilm formation processes. In this study, the *E. coli* K802NR strain (regulated by the *lasI* promoter) was used as a preliminary heterologous screening model to identify potential interference with this pathway. As shown in Figure 2, extracts from *T. clypeatus* R. Heim (Sample #1), *L. squarrosulus* (Sample #4), and *T. porentosus* (Sample #5) significantly inhibited bioluminescence (IF < 0.5) at the highest concentration tested (5 mg/mL).

While this suppression of the *lasI* promoter fusion in an *E. coli* model suggests that these extracts may interfere with signaling mechanisms, these results should be interpreted as preliminary evidence rather than definitive proof of their action in *P. aeruginosa*. Because *lasI* is a *P. aeruginosa*-specific gene and the regulatory context differs significantly between species, this inhibition could alternatively reflect non-specific metabolic interference or suppression of the transcriptional machinery of the *E. coli* host. Nonetheless, the consistency between this preliminary bioreporter screening and the macroscopic antiadhesion efficacy observed against *P. aeruginosa* warrants further investigation. Future validation studies, such as the direct quantification of AHL signaling molecules (e.g., C4-HSL or 3-oxo-C12-HSL) via LC-MS/MS and RT-qPCR analysis of *lasI*, *rhlI*, and *pqsA* gene expression in *P. aeruginosa*, are necessary to confirm the quorum-quenching activities in the target pathogen.

#### 2.6.2. Genotoxicity and DNA Damage

The DPD1718 and DPD2794 strains, controlled by the *recA* promoter, were used as preliminary screening models to detect potential genotoxic stress. *L. squarrosulus* (Sample #4) elicited a pronounced response in the *recA*-regulated DPD1718 strain, achieving induction factors (IF) greater than 3.5 at concentrations ranging from 0.15 to 0.6125 mg/mL (Figure 2).

Because *recA* is an integral component of the SOS repair network, this bioluminescence induction provides a plausible explanation for the genotoxic stress triggered by *L. squarrosulus* in bacterial cells in the present study. However, it is essential to emphasize that these findings in an *E. coli* bioreporter do not guarantee identical DNA-damaging effects in *S. aureus*. The SOS response and DNA repair machinery in Gram-positive *S. aureus* are regulated by distinct molecular mechanisms; therefore, this result serves as a screening-level observation rather than definitive proof of genotoxicity in the target clinical pathogen. Future validation, such as the direct quantification of double-strand breaks via comet assays or γH2AX quantification in *S. aureus*, is required to confirm this genotoxic pathway as the specific driver of the additive antimicrobial synergy observed with gentamicin.

#### 2.6.3. Oxidative Stress Induction

The *katG* promoter in the DPD2511 strain serves as a biosensor for oxidative stress. *V. volvacea* (Sample #2) triggered an extreme, highly specific response, yielding an IF > 7 at a concentration of 5 mg/mL (Figure 2). This indicates the massive generation of reactive oxygen species (ROS) in bacterial cells. Similarly, *T. clypeatus* R. Heim (Sample #1) induced significant oxidative stress (IF ~3.5) at elevated sample concentrations. The massive generation of ROS induced by *V. volvacea* highlights a highly potent but non-selective bactericidal mechanism. While extreme oxidative bursts inflict irreversible damage on bacterial structural proteins, membrane lipids, and nucleic acids to activate the SOS response network [20], this lack of target selectivity directly explains the severe high-dose cytotoxicity observed against mammalian HaCaT cells, as discussed in Section 3.2. This sharply contrasts with the highly selective and safe biocompatibility profile of *T. clypeatus* R. Heim.

#### 2.6.4. Protein Damage and Fatty Acid Biosynthesis Inhibition

Broad-spectrum internal cellular damage was monitored using the TV1061 strain (utilizing the *grpE* promoter for heat shock/protein damage) and DPD2544 strain (utilizing the *fabA* promoter for fatty acid biosynthesis inhibition). Extracts from *T. porentosus* (Sample #5) and *S. commune* (Sample #3) consistently induced both pathways (IF > 1.5) (Figure 2), indicating a broad, non-specific destabilization of the internal protein structures and bacterial cell envelope. Conversely, *T. clypeatus* R. Heim (Sample #1) exerted a sharp inhibitory effect (IF < 0.5) on the TV1061, DPD2544, and K802NR strains at the highest concentration of 5 mg/mL (Figure 2). In the context of whole-cell bioreporter assays, this immediate broad-spectrum suppression at high doses is indicative of acute lethality, as the extract effectively neutralizes bacteria before they possess the physiological capacity to produce a luminescent stress response.

### 2.7. Integrated Metabolomic and Mechanistic Analysis

#### 2.7.1. Chemical Basis for Biocompatibility in *T. fuliginosus* R. Heim

The chemical profiles of the mushrooms were characterized using both positive and negative ionization modes. Positive mode MS analysis revealed an abundance of essential amino acids (arginine and tryptophan), whereas negative mode analysis confirmed the presence of organic acid metabolites, collectively providing a comprehensive view of the regenerative potential of the extract (Table S2 and Figure S1). The highly selective and mitogenic biocompatibility of *T. clypeatus* R. Heim is strongly supported by its enrichment in primary metabolites, specifically essential amino acids and nucleosides (adenosine, guanosine, and inosine). Exogenous purine nucleosides are highly valued in modern tissue engineering. Adenosine is a potent signaling molecule that binds to P1 purinergic receptors on human keratinocytes, actively accelerating wound closure, angiogenesis, and migration of epidermal cells [21]. Furthermore, the high abundance of arginine serves as a critical precursor for localized nitric oxide (NO) synthesis, which is a necessary mediator of collagen deposition during the proliferative phase of wound healing [22].

#### 2.7.2. Antibiofilm and Quorum-Sensing Inhibition in *L. squarrosulus*

The potent antibiofilm efficacy of *L. squarrosulus* appears to be supported by its diverse secondary metabolome. LC-MS profiling identified multiple hydroquinolones, notably 2-heptyl-3-hydroxy-4(1H)-quinolone, which is widely recognized as the native *Pseudomonas* quinolone signal (PQS). PQS is a vital autoregulator that triggers the release of extracellular DNA (eDNA) and pyocyanin, serving as a structural sealant in mature biofilms [23]. The identification of these specific analogs suggests a preliminary hypothesis that the extract may act as a competitive antagonist, potentially binding to the bacterial PqsR receptor and interfering with the pathogen’s ability to construct its EPS matrix.

However, the presence of these compounds does not confirm a definitive mechanism of action, as their functional impact depends on local concentration, bioavailability, and competitive binding affinity. Whether these quinolone analogs act as agonists, partial agonists, or antagonists remains to be elucidated through functional validation. Similarly, while the identification of triterpenoids (e.g., alisol G and ursolic acid) provides a structural basis for membrane-fluidizing hypotheses, the detection of harmol raises the possibility of DNA intercalation; however, these mechanisms remain putative based on compound presence. These findings should be reframed as mechanistic hypotheses, rather than functional proofs. Future studies utilizing functional validation, such as PqsR reporter assays (e.g., *pqsA-lux* fusion), membrane anisotropy studies, or direct gentamicin uptake measurements, are required to substantiate these potential pathways and to distinguish between true quorum-sensing interference and generalized metabolic effects.

Importantly, because our LC-QTOF-MS analysis relied on metabolite annotation without isolating pure compounds, the observed bioactivity cannot be definitively attributed solely to the identified hydroquinolones or triterpenoids. The crude extracts represent complex chemical mixtures, and uncharacterized minor compounds or synergistic multi-component interactions likely contribute significantly to the overall biological effects. Therefore, these causal metabolite-activity relationships should be considered preliminary hypotheses.

#### 2.7.3. Metabolite Profiles of *V. volvacea*, *S. commune*, and *T. porentasus*

*V. volvacea* is characterized by the presence of sugars, acylcarnitines, and the harmala alkaloid harmol. Harmol is a recognized DNA-intercalating agent known to uncouple electron transport chains, which mechanistically corroborates the extreme, non-selective ROS burst (IF > 7) observed in the *katG* bioreporter. The metabolomic signature of *T. porentosus* was heavily dominated by organic acids (malic, succinic, and citric) and long-chain fatty acids. High concentrations of undissociated organic acids readily permeate bacterial cell membranes, lowering the intracellular pH, collapsing the PMF, and triggering widespread denaturation of proteins [24]. This harsh acidic profile strongly correlates with the broad-spectrum structural damage observed in *grpE* bioreporters and its nonselective cytotoxicity toward human keratinocytes.

## 3. Materials and Methods

### 3.1. Mushroom Collection and Extract Preparation

Fruiting bodies of five target species native to Thailand, namely *T. clypeatus* R. Heim (Sample 1), *V. volvacea* (Sample 2), *S. commune* (Sample 3), *L. squarrosulus* (Sample 4), and *T. porentosus* (Sample 5), were collected, dried at 40 °C, and ground into a fine powder (Figure 3). Fruiting bodies, free from blemishes and pieces of soil or substrate, were collected from the Tarad-Thai Market, Thailand, and identified based on their macroscopic morphological characteristics and comparison with local taxonomic guides. Mushroom extracts were prepared by ultrasonic bath extraction of 10 g of dried and ground mushroom powder in 200 mL of 70% ethanol for 30 min, followed by solvent removal using a rotary evaporator Rotavapor^®^ R-300 (BÜCHI Labortechnik AG, Flawil, Switzerland) under vacuum to isolate the bioactive compounds from the extract.

### 3.2. Metabolite Profiling via LC-QTOF-MS

Mushroom extracts (10 mg) were dissolved in 1000 µL of 70% ethanol containing 50 ng/mL sulfadimethoxine as an internal standard. The samples were sonicated for 20 min and centrifuged at 14,000 rpm for 10 min. The supernatant was diluted five-fold with an internal standard solution and analyzed.

Chromatographic separation was performed using an Agilent LC-QTOF 6545XT instrument equipped (Agilent Technologies, Santa Clara, CA, USA) with a Poroshell 120 EC-C18 column (2.1 × 100 mm, 2.7 µm) maintained at 50 °C. The mobile phase consisted of 0.1% formic acid in water (A) and 0.1% formic acid in acetonitrile (B) at a flow rate of 0.4 mL/min. The gradient elution program was set as follows: 0–0.5 min, 0% B; 0.5–10.5 min, 55% B; 10.5–12.5 min, 75% B; 12.5–14.0 min, 100% B, followed by column equilibration [25].

Mass spectrometry was performed in the high-resolution positive and negative electrospray ionization modes, respectively. The source parameters were strictly optimized as follows: drying gas temperature of 325 °C, drying gas flow of 13 L/h, sheath gas temperature of 275 °C, sheath gas flow of 12 L/h, and nebulizer pressure of 45 psi [26]. The capillary voltages were set at 4000 V (positive) and 3000 V (negative), with collision energies of 20 eV (positive) and 10 eV (negative). The MS acquisition parameters included an acquisition rate of 3.35 spectra/s, a maximum of 10 precursors per cycle, a precursor threshold of 5000 counts, and a retention-time threshold of 0.001%. The MS1 and MS2 mass ranges were 40–1700 *m*/*z* and 25–1000 *m*/*z*, respectively, with an isotope width of 1.3 *m*/*z*. Mass accuracy was maintained via internal reference mass correction using: pos 121.0509 m/z and 922.0098 *m*/*z*; neg 112.9856 m/z and 1033.9881 *m*/*z*. Data processing, including alignment, normalization, and feature filtration, was performed using MS-Dial (version 5.3) against the Fiehn/Vaniya natural product database.

### 3.3. Biocompatibility and Cell Viability (CCK-8 Assay)

To confirm the safety of the extracts for biomedical applications, cell viability was evaluated using the Cell Counting Kit-8 (CCK-8) assay (Dojindo Laboratories, Kumamoto, Japan) on human HaCaT keratinocyte cells. Cells were cultured under standard conditions and treated with mushroom extracts at final concentrations ranging from 2 to 2000 µg/mL. The dried extracts were dissolved in dimethyl sulfoxide (DMSO) to prepare stock solutions, which were subsequently diluted with the culture medium to the desired concentrations, with the final DMSO concentration maintained at a non-cytotoxic level. Following treatment, CCK-8 reagent containing WST-8 was added, and absorbance was measured at 450 nm using a microplate reader (Thermo Scientific Multiskan Sky Microplate Spectrophotometer, Thermo Fisher Scientific, Waltham, MA, USA). The amount of water-soluble formazan dye produced is directly proportional to the number of metabolically active and viable cells [27].

### 3.4. Antimicrobial Activity and Checkerboard Assay

The minimum inhibitory concentration (MIC) and minimum bactericidal concentration (MBC) of the extracts were determined using microdilution assays. Briefly, 100 µL of the stock extract solution prepared in 0.25% DMSO was mixed with 50 µL of Mueller-Hinton liquid medium in 96-well microplates, followed by serial dilution. Subsequently, 50 µL of a bacterial suspension (adjusted to 1 × 10^5^ cfu/mL) was mixed with triphenyl tetrazolium chloride (TTC) at a final concentration of 0.05% as a redox indicator, which turns pink in the presence of metabolically active (viable) bacteria, and incubated at 37.0 ± 0.5 °C for 24.0 ± 0.5 h. The MIC was defined as the lowest concentration that did not exhibit a pink color, indicating successful suppression of bacterial respiration and growth. To determine the MBC, the contents of the clear wells were inoculated onto fresh Mueller-Hinton fresh agar plates and incubated at 37.0 ± 0.5 °C for an additional 24.0 ± 0.5 h; the MBC was recorded as the lowest concentration yielding no visible bacterial colonies [28].

To evaluate the synergistic interactions between the extracts and conventional antibiotics, a checkerboard microdilution assay was performed against *P. aeruginosa* ATCC 35032, *S. aureus* ATCC 25923, *E. coli* ATCC 25922, *Enterococcus faecalis* ATCC 29219, and *Salmonella enteritidis* ATCC 22076. Mushroom extracts (0.156–20 mg/mL) and gentamicin (0.0039–0.5 mg/mL) were combined in matrices with varying concentrations. The interactive effect was quantified by calculating the Fractional Inhibitory Concentration Index (FICI) using the following equation:

(1) $$FICI=\frac{{MIC}_{X,comb}}{{MIC}_{X,alone}}+\frac{{MIC}_{Y,comb}}{{MIC}_{Y,alone}}$$

where MIC_X,comb_ and MIC_Y,comb_ represent the MIC values of the extract and antibiotic when used in combination, and MIC_X,alone_ and MIC_Y,alone_ represent their respective MIC values when tested separately.

### 3.5. Antiadhesion and Antibiofilm Assays

The ability of the extracts to inhibit bacterial adhesion and subsequent biofilm formation was assessed using crystal violet staining in flat-bottomed transparent polystyrene 96-well microplates. For the antiadhesion assay, wells containing mushroom extracts diluted in Mueller–Hinton broth supplemented with 0.25% DMSO were inoculated with 50 µL of bacterial culture and incubated at 37 °C for 24 h. To assess antibiofilm formation activity, the same inoculation protocol was followed, but the incubation period was extended to 48 h to allow mature biofilm establishment [19].

Following the respective incubation periods, the microplates were washed thrice with sterile water to remove planktonic cells and were dried. Adherent cells or established biofilms were stained with 0.1 mL of 1% crystal violet solution for 15 min. After washing off the excess dye and drying the plates, the bound crystal violet was solubilized using acetic acid (33%) or ethanol (96%). The absorbance was measured at 590 nm using a microplate reader Varioskan (Thermo Fisher Scientific, Waltham, MA, USA). The percentage of adhesion inhibition (%IA) was calculated using the following formula:

(2) $$\%IA=\;\left(\frac{A_{K}-A_{S}}{A_{K}}\right)\times 100$$

where *A_K_* is the average absorbance of the untreated control samples, and *A_S_* is the average absorbance of the extract-treated samples.

### 3.6. Mechanistic Screening via Bioluminescent Whole-Cell Bioreporter Panel

To rapidly elucidate the specific molecular mechanisms of antimicrobial action, a comprehensive panel of six genetically modified *E. coli* bioluminescent reporter strains was used. The selected strains and their respective promoter sensitivities were as follows: K802NR (promoter *lasI* for QS disruption), TV1061 (promoter *grpE* for heat shock and protein damage), DPD2794 and DPD1718 (promoter *recA* for genotoxicity and DNA damage), DPD2544 (promoter *fabA* for fatty acid biosynthesis inhibition), and DPD2511 (promoter *katG* for oxidative stress) [8].

The bacterial strains were cultivated on Luria–Bertani (LB) agar supplemented with the appropriate antibiotics (kanamycin sulfate or ampicillin) and incubated at 37 °C. Prior to screening, secondary cultures were grown in antibiotic-free LB medium to an optical density of 0.2 at 600 nm, corresponding to the optimal exponential growth phase of the bacteria. The bioassay was performed in white opaque 96-well microtiter plates containing 90 µL of the active bacterial culture and 10 µL of diluted mushroom extract. Double-distilled water and 0.25% DMSO served as negative controls, while established chemical inducers (nalidixic acid, doxorubicin, methanol, ethanol, and phenol) served as positive controls.

Bioluminescence kinetics were recorded using a Luminoskan Ascent luminometer (Thermo Fisher Scientific, Waltham, MA, USA) over a 20 h period at 5 min intervals. The cellular response to the extracts was quantified by calculating the Induction Factor (IF):

(3) $$IF=\frac{{Luminescence}_{sample,max}}{{Luminescence}_{control,max}}$$

where L_sample,max_ is the maximum bioluminescence generated by the treated sample and L_control,max_ is the maximum bioluminescence generated by the negative control.

### 3.7. Statistical Analysis

All quantitative experiments were conducted in at least three independent biological replicates (*n* = 3), with each biological replicate consisting of three technical replicates. Data are expressed as the mean ± standard deviation (SD). Statistical significance was evaluated using one-way analysis of variance (ANOVA), followed by Dunnett’s post-hoc test to compare multiple treatment groups with the control group. To account for the inflation of type I error rates inherent in multiple comparisons, a Bonferroni correction was applied to all *p*-value thresholds. Differences were considered statistically significant at *p* < 0.05, *p* < 0.01, and *p* < 0.001. All analyses were performed using IBM SPSS Statistics software (version 26.0). While sample sizes were determined based on established protocols for in vitro microbiological and cell-based assays, future studies should include a formal a priori power analysis to further optimize the experimental design and ensure sufficient sensitivity to detect moderate effect sizes.

### 3.8. Limitations and Future Directions

While the baseline antimicrobial and antioxidant properties of tropical macrofungi, such as *Lentinus* spp. and *Schizophyllum* spp., are well documented in the existing literature, earlier investigations have predominantly relied on fundamental phenotypic evaluations, such as disk diffusion or standard MIC assays. Consequently, a significant knowledge gap persists regarding the precise molecular pathways driving these biological effects. By integrating untargeted LC-QTOF-MS metabolomic profiling with a high-throughput panel of genetically modified bioluminescent *E. coli* bioreporters, our findings advance the field beyond basic bactericidal screening. This combined platform allows for the preliminary mapping of macroscopic antibiofilm behaviors to specific molecular stress responses in bacteria. For example, the antibiofilm efficacy of *L. squarrosulus* may be correlated with specific stress pathways, such as potential quorum-sensing interference and genotoxic stress, supported by the identification of hydroquinolones and triterpenoids. Furthermore, whereas many crude botanical and fungal extracts in the literature exhibit dose-limiting, non-specific toxicity, the distinct mitogenic effect of *T. clypeatus* R. Heim on human keratinocytes separates its biological profile from that of general fungal extracts. This highlights a highly specific, previously underexplored regenerative profile that supports further targeted investigation of topical applications.

However, it is important to note that while our high-throughput bioluminescent *E. coli* bioreporter panel provides valuable preliminary insights into potential quorum-sensing disruption and stress-induction mechanisms, it relies on heterologous models. The regulatory networks in the target pathogens differ substantially from those in *E. coli*. To definitively validate these mechanistic hypotheses, future studies must evaluate the direct transcriptional response of specific clinical strains. Specifically, quantitative real-time PCR (RT-qPCR) should be employed to measure the expression of key quorum-sensing regulatory genes, including *lasI*, *lasR*, *rhlI*, and *pqsA* in *P. aeruginosa*, as well as *agrA* and RNAIII expression in *S. aureus*. These targeted molecular validations are essential to confirm whether the bioactive compounds in *L. squarrosulus* disrupt specific virulence pathways in clinical pathogens.

## 4. Conclusions

This study systematically evaluated the biomedical potential of ethanolic extracts from five native Thai mushroom species, revealing distinct biological activities associated with their specific metabolomic profiles. Through comprehensive biological and chemical evaluations, two standout candidates emerged with the potential for distinct biomedical applications. *T. clypeatus* R. Heim exhibited exceptional biocompatibility, proving to be the safest and most effective extract for promoting human keratinocyte (HaCaT) proliferation across a wide concentration range without inducing cytotoxicity. Its rich primary metabolomic profile, abundant in essential amino acids and nucleosides, suggests its potential for topical wound-healing applications, although critical endpoints, such as cell migration and re-epithelialization, must be validated in future studies. In contrast, *L. squarrosulus* exhibited strong antibiofilm properties and moderate direct antimicrobial activity, particularly against *S. aureus* and *P. aeruginosa*. It exhibited a weak additive effect when combined with the conventional antibiotic gentamicin. Mechanistic bioreporter screening and LC-QTOF-MS profiling suggested potential quorum-sensing disruption and genotoxic stress, although these specific pathways and the exact contributions of the annotated hydroquinolones remain unconfirmed in the target clinical pathogens and require further validation. While *S. commune* showed moderate and plateauing biological effects, *V. volvacea* and *T. porentosus* induced unfavorable high-dose cytotoxicity linked to severe oxidative stress and high organic acid concentrations. These profiles indicate that they are less suitable for direct biomedical applications in human tissues at high concentrations. Ultimately, *T. clypeatus* R. Heim and *L. squarrosulus* represent highly promising and mechanistically explored natural resources. Future in vivo studies should prioritize harnessing *T. clypeatus* R. Heim for tissue regeneration and isolating specific bioactive fractions of *L. squarrosulus* to explore their viability as adjunctive agents against biofilm-associated diseases.

## Figures and Tables

**Figure 1:**
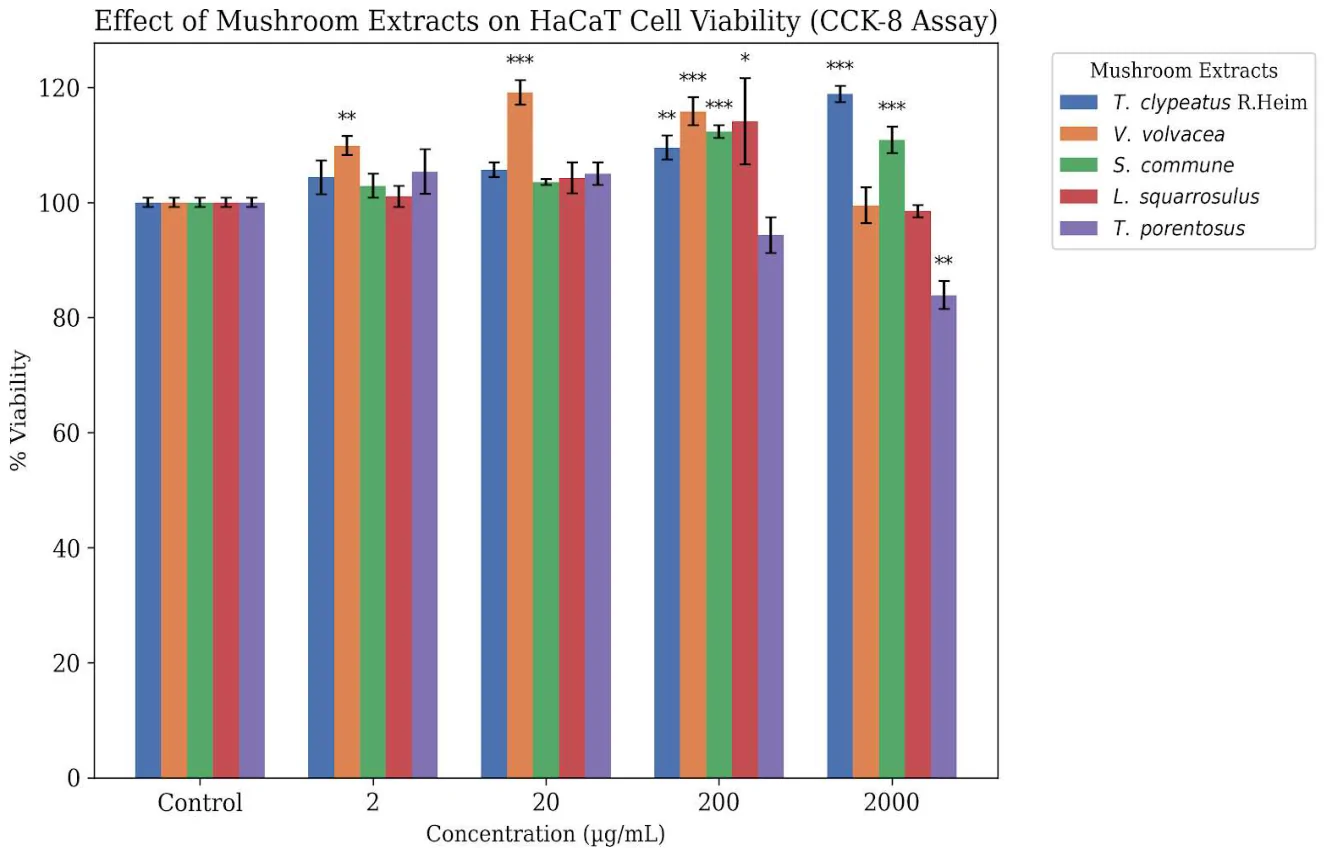
Effect of five Thai mushroom ethanolic extracts on HaCaT human keratinocyte viability. Cells were treated with 2, 20, 200, and 2000 µg/mL of the extracts for 24 h, and cell viability was assessed using the CCK-8 assay. Data are expressed as the mean ± standard deviation (SD). Statistical significance was determined using One-way ANOVA compared to the untreated control (* *p* < 0.05, ** *p* < 0.01, *** *p* < 0.001).

**Figure 2:**
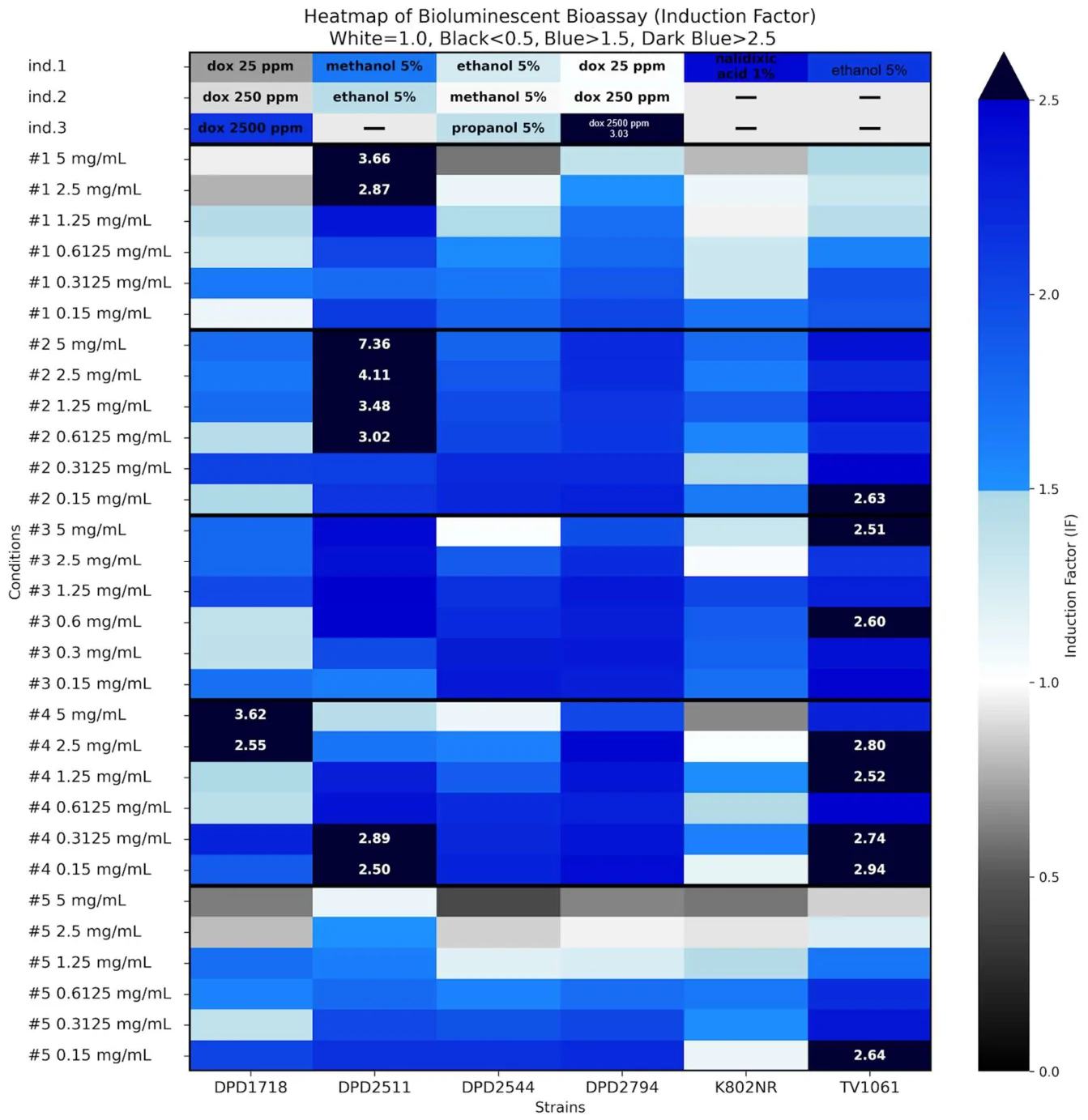
Heatmap illustrating the comparative Induction Factors across the full bioreporter panel. # represents the sample number.

**Figure 3:**
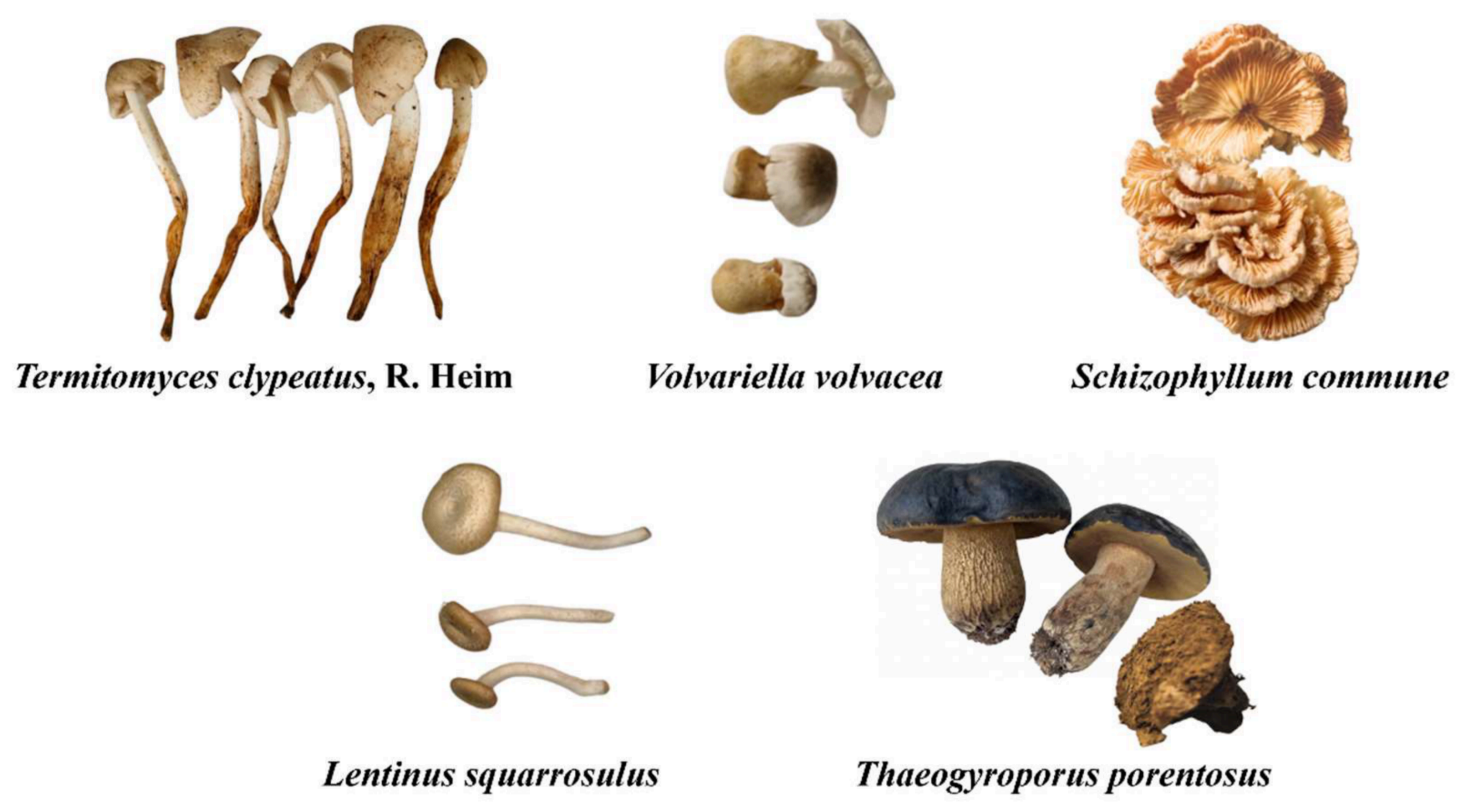
Fruiting bodies of five mushroom species native to Thailand: *T. clypeatus* R. Heim (Sample 1), *V. volvacea* (Sample 2), *S. commune* (Sample 3), *L. squarrosulus* (Sample 4), and *T. porentosus* (Sample 5) were used.

**Table 1:** Antimicrobial Activity (MIC and MBC values, mg/mL) of ethanolic extracts from five Thai mushroom species.

Bacterial Strain	*S. aureus* (ATCC 25923)	*E. faecalis* (ATCC 29219)	*E. coli* (ATCC 25922)	*S. enteritidis* (ATCC 22076)	*P. aeruginosa* (ATCC 35032)
mg/mL	MIC/MBC	MIC/MBC	MIC/MBC	MIC/MBC	MIC/MBC
*T. clypeatus* R. Heim	-/-	-/-	-/-	20/-	-/-
*V. volvacea*	10/-	20/-	20/-	20/-	-/-
*S. commune*	20/-	-/-	-/-	-/-	-/-
*L. squarrosulus*	2.5/20	-/-	-/-	20/-	10/-
*T. porentosus*	20/-	-/-	-/-	-/-	-/-
Gentamicin	<0.0078/0.0156	<0.0078/0.5	<0.0078/0.125	<0.0078/0.0078	<0.0078/0.0078
Penicillin	<0.0078/0.0625	-/-	0.25/0.25	0.0156/0.031	-/-

Note: MIC, minimum inhibitory concentration; MBC, minimum bactericidal concentration. - indicates that the sample did not exhibit any antimicrobial activity within the tested range.

**Table 2:** Fractional Inhibitory Concentration Index (FICI) of Mushroom Extracts Combined with Gentamicin Against *S. aureus* ATCC 25923.

Mushroom Species	FICI Value	Interaction Outcome
*T. clypeatus* R. Heim	2.034	Indifference
*V. volvacea*	2.034	Indifference
*S. commune*	4.002	Indifference
*L. squarrosulus*	0.832	Additive effect
*T. porentosus*	2.065	Indifference

**Table 3:** Summary of maximum bacterial adhesion inhibition (%IA) and optimal concentration (mg/mL).

Bacteria/Mushroom	*T. clypeatus* R. Heim	*V. volvacea*	*S. commune*	*L. squarrosulus*	*T. porentosus*
*E. coli*	100.0% (1.25, 0.625)	92.6% (0.625)	74.1% (0.625)	25.9% (0.625)	74.1% (1.25)
*E. faecalis*	93.9% (10)	0.0%	49.0% (1.25)	81.6% (20)	100.0% (5)
*P. aeruginosa*	100.0% (10, 5, 2.5)	77.3% (20)	86.4% (5, 2.5)	81.8% (20, 10)	27.3% (20)
*S. aureus*	0.0%	100.0% (0.625)	100.0% (0.625)	100.0% (1.25, 0.625)	16.7% (20, 0.625)
*S. enteritidis*	0.0%	0.0%	96.0% (0.625)	92.6% (0.625)	0.0%

The values represent the maximum percentage of adhesion inhibition observed (%IA). The numbers in parentheses denote the extract concentration (mg/mL) at which maximum inhibition was achieved.

## Data Availability

All data produced in this study are available in the manuscript. Additionally, key bioactive metabolites identified via LC-QTOF-MS and their correlated biological activities are provided in the Supplementary Materials.

## Supplementary Materials

The following supporting information can be downloaded at: https://www.mdpi.com/article/10.3390/molecules31193457/s1, Table S1: Key Bioactive Metabolites Identified via LC-QTOF-MS and Their Correlated Biological Activities, Table S2: LC-QTOF-MS parameters and identification data for bioactive compounds detected in mushroom extracts (Positive and Negative Ionization Modes), Figure S1: (a) Total Ion Chromatogram (TIC) of mushroom extracts in positive ionization mode. S1b is the Total Ion Chromatogram (TIC) of mushroom extracts in negative ionization mode, and Table S3: Percentage of Bacterial Adhesion Inhibition (%IA) by the Five Mushroom Ethanolic Extracts.
