# Supplementary material for: Integrated Metabolomic and Bioreporter Screening of Five Thai Mushroom Extracts: Exploring Antibiofilm and Biocompatibility Profiles
Source: Molecules. 2026 Sep 28;31(19):3457. doi: 10.3390/molecules31193457 (PMC13666336; doi:10.3390/molecules31193457)
Supplement: Supplementary file 1 [file molecules-31-03457-s001.zip › Supporting Information - revised.pdf]

Supplementary Information (SI)

Table S1. Key Bioactive Metabolites Identified via LC-QTOF-MS and Their Correlated Biological Activities.

Mushroom Species	Dominant Metabolite Classes	Key Identified Compounds	Correlated Biological Activity
Termitomyces fuliginosus	Primary metabolites (Amino acids, Nucleosides)	Arginine, Phenylalanine, Tryptophan, Adenosine, Guanosine, Inosine	Exceptional biocompatibility; provides essential nutrients that strongly promote HaCaT human keratinocyte proliferation without cytotoxicity.
Volvariella volvacea	Sugars, Acylcarnitines, Harmala alkaloids	Hexanoyl-L-carnitine, Butyryl carnitine, Harmol	Induced severe oxidative stress in bioluminescent *E. coli*; exhibited biphasic cytotoxic effects on HaCaT cells at higher concentrations.
Schizophyllum commune	Alkaloids, Tryptamines, Phenethylamines, Phospholipids	Sparteine, Oxymatrine, Pilocarpine, LPC 18:2, LPE 18:2	Demonstrated moderate, dose-dependent antiadhesion and antibiofilm properties driven by a complex mixture of diverse secondary metabolites.
Lentinus squarrosulus	Hydroquinolones, Triterpenoids, Cinnamic acid	2-heptyl-3-hydroxy-4(1H)-quinolone (*Pseudomonas* quinolone signal), Alisol G, Ursolic acid	Potent antibiofilm efficacy and additive antimicrobial synergism; quinolone analogs actively disrupt bacterial quorum-sensing communication networks.
Thaeogyroporus porentosus	Organic acids, Long-chain fatty acids	Malic acid, Succinic acid, Citric acid, Palmitic acid, Oleic acid	High concentrations of organic acids correlate with broad-spectrum bacterial inhibition, direct lethality (fatty acid biosynthesis inhibition), and high-dose cytotoxicity.

Table S2. LC-QTOF-MS parameters and identification data for bioactive compounds detected in mushroom extracts (Positive and Negative Ionization Modes; a summarized list, complete data can be found in Excel file Table S2 Supplementary Materials).

Metabolite Name	Bioactive Class & Correlated Biological Activity	Average RT (min)	Average m/z	Ionization Mode
Pseudomonas quinolone signal (PQS)	Hydroquinolone: Competitively disrupts P. aeruginosa quorum-sensing and extracellular matrix formation	10.390	260.1641	Positive
Ursolic Acid	Triterpenoid: Antimicrobial agent, potentially interacts with bacterial lipid bilayers	8.121	479.3457	Positive
Alisol G	Triterpenoid: Antimicrobial secondary metabolite	6.063	495.3407	Positive
Harmol	Harmala Alkaloid: DNA-intercalating agent known to trigger massive, non-selective ROS bursts	4.149	199.0867	Positive
Adenosine	Nucleoside: Potent signaling molecule that accelerates wound closure via P1 purinergic receptors	0.698	268.1043	Positive
Guanosine	Nucleoside: Mitogenic primary metabolite supporting human keratinocyte (HaCaT) proliferation	2.451	282.0853	Negative
Inosine	Nucleoside: Primary metabolite utilized in modern tissue engineering	2.439	267.0739	Negative
Arginine	Essential Amino Acid: Critical precursor for localized nitric oxide (NO) synthesis and collagen deposition	0.645	175.1191	Positive
Tryptophan	Essential Amino Acid: Supports regenerative and proliferative biological profiles	3.380	205.0968	Positive
Succinic Acid	Organic Acid: Readily permeates cell membranes, lowering intracellular pH and causing protein denaturation	1.282	117.0187	Negative
Malic Acid	Organic Acid: Contributes to broad-spectrum structural damage and high-dose cytotoxicity	0.696	133.0139	Negative
Citrate (Citric Acid)	Organic Acid: Drives severe genotoxic/oxidative stress responses at high concentrations	0.687	191.0190	Negative

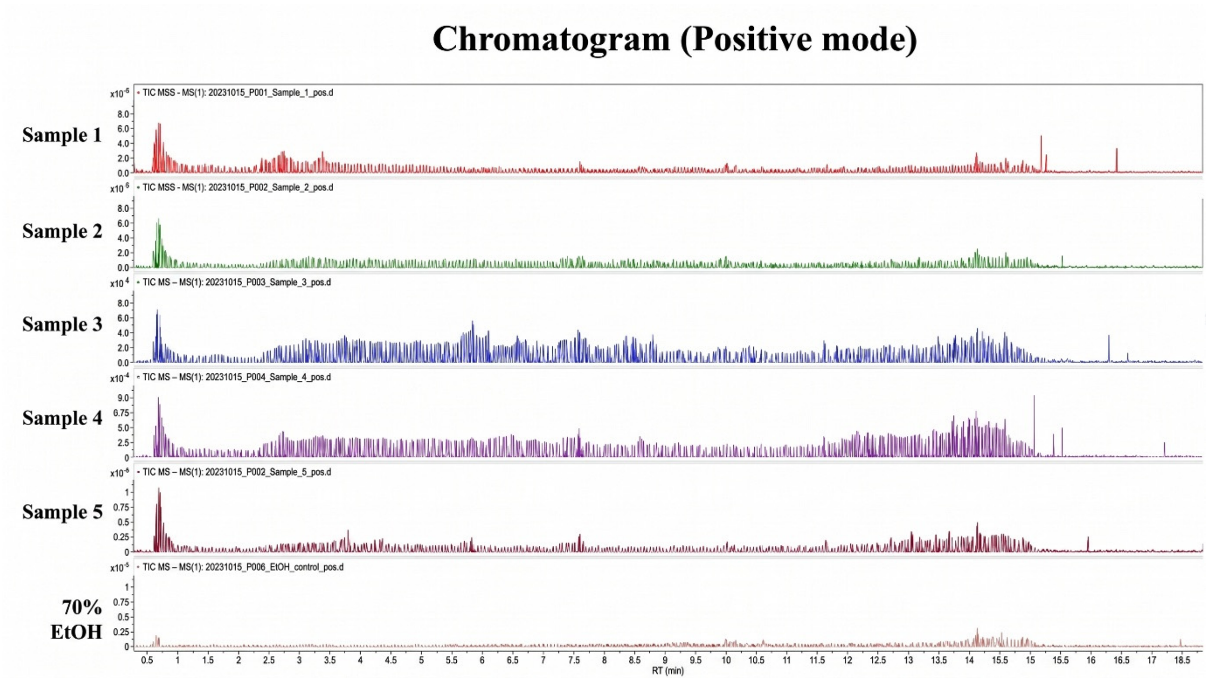

(a)

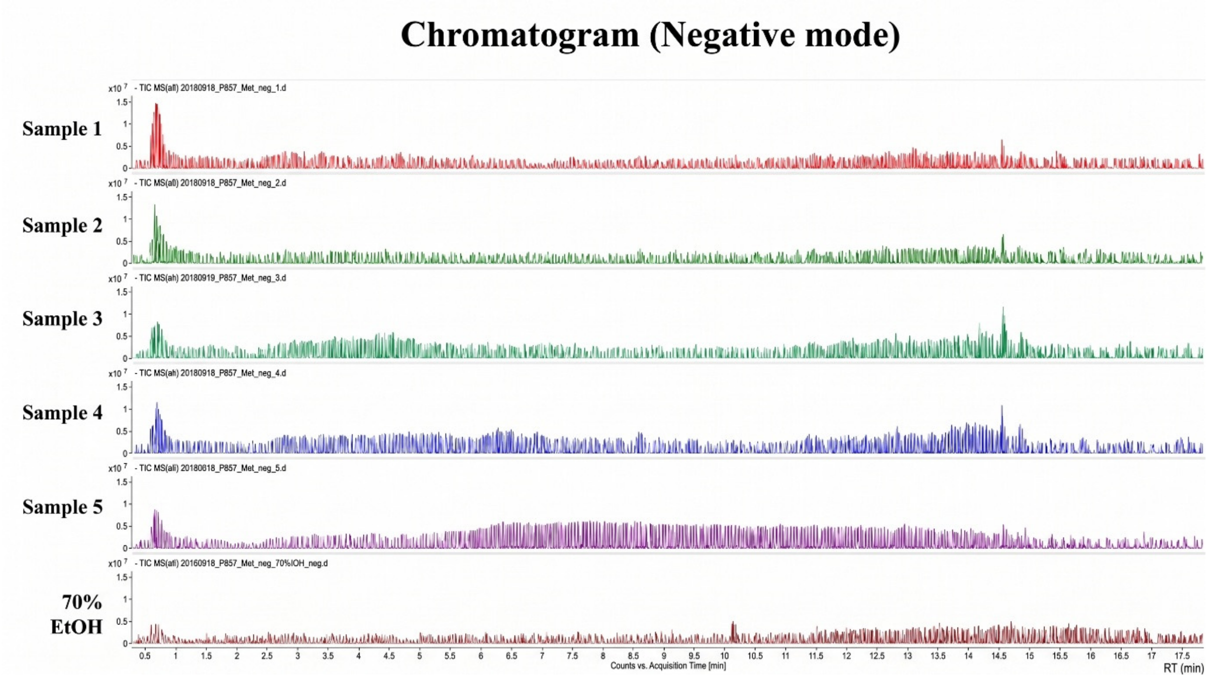

(b)

Figures S1. (a) Total Ion Chromatogram (TIC) of mushroom extracts in positive ionization mode. S1b is the Total Ion Chromatogram (TIC) of mushroom extracts in negative ionization mode.

Table S3. Percentage of Bacterial Adhesion Inhibition (%IA) by the Five Mushroom Ethanolic Extracts

**Test Microorganism**	**Concentration (mg/mL)**	***T. clypeatus* R. Heim**	***V. volvacea***	***S. commune***	***L. squarrosulus***	***T. porentosus***
E. coli (ATCC 25922)	20	29.6 ± 1.1	0.0 ± 0.0	0.0 ± 0.0	0.0 ± 0.0	44.4 ± 4.5
	10	33.3 ± 6.4	0.0 ± 0.0	0.0 ± 0.0	0.0 ± 0.0	50.4 ± 3.1
	5	37.0 ± 3.8	0.0 ± 0.0	3.7 ± 1.8	0.0 ± 0.0	59.3 ± 2.4
	2.5	40.7 ± 2.9	0.0 ± 0.0	11.1 ± 4.3	0.0 ± 0.0	66.7 ± 3.8
	1.25	100.0 ± 0.0	48.1 ± 4.1	59.3 ± 2.9	0.0 ± 0.0	74.1 ± 1.0
	0.625	100.0 ± 0.0	92.6 ± 3.2	74.1 ± 3.9	25.9 ± 3.1	59.3 ± 3.6
Enterococcus faecalis (ATCC 29219)	20	79.6 ± 1.8	0.0 ± 0.0	40.8 ± 2.2	81.6 ± 3.4	73.5 ± 4.1
	10	93.9 ± 2.2	0.0 ± 0.0	42.9 ± 5.1	65.3 ± 3.7	95.9 ± 2.7
	5	89.8 ± 1.6	0.0 ± 0.0	46.9 ± 3.7	53.1 ± 5.1	100.0 ± 0.0
	2.5	83.7 ± 4.2	0.0 ± 0.0	46.9 ± 3.5	59.2 ± 2.3	61.2 ± 1.8
	1.25	53.1 ± 2.4	0.0 ± 0.0	49.0 ± 2.9	55.1 ± 3.5	18.4 ± 2.4
	0.625	63.3 ± 2.1	0.0 ± 0.0	6.1 ± 2.4	53.7 ± 1.9	0.0 ± 0.0
P. aeruginosa (ATCC 35032)	20	0.0 ± 0.0	77.3 ± 3.3	81.9 ± 6.3	81.8 ± 4.2	27.3 ± 2.1
	10	100.0 ± 0.0	72.7 ± 2.7	77.3 ± 3.1	81.8 ± 5.1	24.5 ± 1.5
	5	100.0 ± 0.0	50.0 ± 3.1	86.4 ± 3.5	59.1 ± 3.6	18.2 ± 1.1
	2.5	100.0 ± 0.0	50.0 ± 1.7	86.4 ± 2.8	36.4 ± 3.2	0.0 ± 0.0
	1.25	72.7 ± 4.1	50.0 ± 1.4	77.3 ± 4.3	4.5 ± 1.9	0.0 ± 0.0
	0.625	63.6 ± 1.6	18.2 ± 2.1	22.7 ± 4.9	0.0 ± 0.0	0.0 ± 0.0
S. aureus (ATCC 25923)	20	0.0 ± 0.0	0.0 ± 0.0	0.0 ± 0.0	16.7 ± 0.7	0.0 ± 0.0

	10	0.0 ± 0.0	0.0 ± 0.0	0.0 ± 0.0	33.3 ± 1.7	0.0 ± 0.0
	5	0.0 ± 0.0	0.0 ± 0.0	0.0 ± 0.0	54.3 ± 1.4	0.0 ± 0.0
	2.5	0.0 ± 0.0	0.0 ± 0.0	9.3 ± 1.8	83.3 ± 3.8	0.0 ± 0.0
	1.25	0.0 ± 0.0	83.3 ± 2.2	50.0 ± 2.6	100.0 ± 0.0	0.0 ± 0.0
	0.625	0.0 ± 0.0	100.0 ± 0.0	100.0 ± 0.0	100.0 ± 0.0	16.7 ± 0.9
Salmonella enteritidis (ATCC 22076)	20	0.0 ± 0.0	0.0 ± 0.0	0.0 ± 0.0	0.0 ± 0.0	0.0 ± 0.0
	10	0.0 ± 0.0	0.0 ± 0.0	14.3 ± 1.2	18.1 ± 1.1	0.0 ± 0.0
	5	0.0 ± 0.0	0.0 ± 0.0	18.0 ± 1.9	26.0 ± 1.3	0.0 ± 0.0
	2.5	0.0 ± 0.0	0.0 ± 0.0	34.6 ± 0.9	34.0 ± 1.7	0.0 ± 0.0
	1.25	0.0 ± 0.0	0.0 ± 0.0	88.4 ± 1.9	42.2 ± 2.1	0.0 ± 0.0
	0.625	0.0 ± 0.0	0.0 ± 0.0	96.0 ± 3.6	92.6 ± 1.8	0.0 ± 0.0

All measurements are expressed as the mean value +/- the standard deviation of 6 repetitions.
